# Broadening Participation in the Society for Integrative and Comparative Biology

**DOI:** 10.1093/icb/icx004

**Published:** 2017-07-24

**Authors:** Cheryl A.D. Wilga, Michele Nishiguchi, Brian Tsukimura

**Affiliations:** * Biological Sciences Department, University of Alaska Anchorage, 3101 Science Circle, Anchorage, AK 99508, USA; † Department of Biology, New Mexico State University, PO Box 30001, MSC 3AF, Las Cruces, NM 88003, USA; ‡ Department of Biology, Fresno, 2555 E. San Ramon Avenue, CA 93740, USA

## Abstract

The goal of the Society for Integrative and Comparative Biology’s Broadening Participation Committee (SICB BPC) is to increase the number of underrepresented group (URG) members within the society and to expand their capabilities as future researchers and leaders within SICB. Our short-term 10-year goal was to increase the recruitment and retention of URG members in the society by 10%. Our long-term 25-year goal is to increase the membership of URG in the society through recruitment and retention until the membership demographic mirrors that of the US Census. Our plans to accomplish this included establishment of a formal standing committee, establishment of a moderate budget to support BPC activities, hosting professional development workshops, hosting diversity and mentor socials, and obtaining grant funds to supplement our budget. This paper documents broadening participation activities in the society, discusses the effectiveness of these activities, and evaluates BPC goals after 5 years of targeted funded activities. Over the past 5 years, the number of URG members rose by 5.2% to a total of 16.2%, members who report ethnicity and gender increased by 25.2% and 18%, respectively, and the number of members attending BPC activities has increased to 33% by 2016. SICB has made significant advances in broadening participation, not only through increased expenditures, but also with a commitment by its members and leadership to increase diversity. Most members realize that increasing diversity will both improve the Society’s ability to develop different approaches to tackling problems within integrative biology, and help solve larger global issues that are evident throughout science and technology fields. In addition, having URG members as part of the executive committee would provide other URG members role models within the society, as well as have a voice in the leadership that represents diversity and inclusion for all scientists.

## Introduction

The Society for Integrative and Comparative Biology (SICB) is one of the largest and most prestigious professional associations of its kind. The Society is dedicated to promoting the pursuit and public dissemination of information relating to biological sciences. SICB takes pride in the fact that one of the Society’s focal points is to support student members, and that the organization is fundamentally committed to the advancement and development of early career investigators through its programs, meetings, and journal publications (Integrative and Comparative Biology).

The main goal of SICB’s Broadening Participation Committee (BPC) is to increase the number of underrepresented group (URG) members within SICB and to expand their capabilities as future researchers within the SICB divisions. SICB has a longstanding mission and commitment to increasing the diversity of URG members, and recognizes the importance of engaging this group of scientists to address future needs within various biological disciplines. Integration among a number of different and diverse disciplines is crucial for our understanding of complex biological questions, and the capability of enabling our future scientists to tackle innovative and interesting ideas regarding organismal variation is in part driven by approaching questions from unique backgrounds and experiences (Schmidt 2010; Kendall 2011). SICB’s Broadening Participation activities provide that platform to nurture students, postdoctoral researchers, and beginning young investigators, which will create a community that will enhance their participation in the society and beyond (Ely and Thomas 2001). We hope that our objectives will equip these young scientists with the toolkit needed to be successful within their careers and eventually become leaders in their fields, utilizing their background and experiences to become our future innovators and teachers (Goode 2004).

The BPC proposed a 5-year Strategic Plan in 2010 to broaden participation within the Society. The proposed objectives were aimed at coalescing and enhancing the experience of participants with new activities that complement those already in place. We established a program that will develop and increase participation of URG (URGs = including minorities, those with disabilities, first generation college attendees, and veterans) by publicizing the benefits of being an active SICB member and encouraging them to participate in leadership roles (Gardner 2013). Our short-term 10-year goal was to increase the recruitment and retention of URG members in the society by 10%. Our long-term 25-year goal is to increase the membership of URG in the society through recruitment and retention until the membership demographic mirrors that of the US Census. Our plans to accomplish this included establishment of a formal standing committee, establishment of a budget to support BPC activities, offering a variety of professional development workshops, hosting diversity socials and obtaining grant funds to supplement our budget. This paper documents broadening participation activities in SICB, discusses the effectiveness of these activities and assesses BPC goals after 5 years of targeted activities.

### The importance of diversity in science

There is a deficit of URG members in scientific societies, which is likely due to the deficit of URGs graduating from universities (Smith 1991; Xu and Martin 2011). Diverse learning environments are most effective with all members benefiting from the increased awareness and broader perspectives of its members (Gurin et al. 2004; Peckham et al. 2007; Holly 2013). Thus, groups involving diverse members provide greater critical analyses of problems and solve them in more innovative ways (McLeod et al. 1996). If the trend in the United States showing an increased URG population continues, the majority of children born in the 21st century will belong to URGs, which are underrepresented in STEM fields (NSF 2005). To be responsive to the rapidly changing demographics of the United States, there is a critical need to broaden participation of URGs in STEM fields, such as those represented in SICB. To be effective in promoting the pursuit and dissemination of relevant and timely biological information to the public, membership demographics in SICB must reflect that of society.

### Early history of broadening participation in SICB

There are several SICB committees that provide opportunities for students at annual meetings prior to 2002 through the present. The Student/Postdoctoral Affairs Committee hosts a Student First Timer workshop explaining how to get the most out of your SICB meeting, and a topical workshop at each annual meeting. The Student Support Committee is charged with overseeing activities related to student support, including the Charlotte Mangum awards that cover housing or registration, as well as individual research awards (Grants-In-Aid-of-Research). The Education Council arranges for undergraduate students to display their posters near the plenary session at annual meetings. However, until 2002 there were no efforts at increasing membership of URGs. Then-President Marvalee Wake created the BPC in 2002, with the goal of increasing diversity in SICB and fields of Integrative and Comparative Biology. From 2005 until 2009, local faculty and their undergraduate students, along with high school students and their teachers, were recruited to attend and present posters at the annual meeting. Registration and lodging were provided for up to 10 individuals each year. SICB graduate student members were then recruited to mentor the local undergraduate and high school students throughout the meeting. A complimentary breakfast was held on the first day of the meeting for self-identified ethnic minorities to network, and where National Science Foundation program directors spoke about funding opportunities. These were good attempts to introduce science in the SICB to local URG undergraduate and high school students and faculty, but it was not clear how many of these local recruits remained members and attended future annual meetings. The Then-BPC Chair Patricia Hernandez initiated a mechanism for self-identification of ethnicity and gender on annual meeting registration forms for the 2009 annual meeting and the 2010 annual membership renewal. These data continue to be collected on annual (online) membership forms as well as for annual conference attendees.

### Formalizing the BPC in SICB

The Executive Committee recognized the need for greater diversity and in 2009 formally recognized the BPC as a standing committee of the SICB where the Chair was elevated to the SICB Executive Committee, which permits participation in voting on SICB activities, especially in budget discussions. This enabled the BPC, which included the authors (Cheryl Wilga, Then-BPC Chair, Brian Tsukimura, Then-Program Officer and Then-BPC member, and Nish Nishiguchi, The-BPC Member), to develop a regular budget ($10,000 in 2010 that was gradually increased to $15,000 in 2012). SICB students indicated a need for financial support to attend the annual meeting; therefore, the BPC established travel awards to allow more URG members to attend the annual meeting. The BPC started offering two workshops geared toward the needs of URG to provide professional development opportunities. One of these workshops focused on junior members (graduate students) and the other targeted senior members (postdocs and faculty). The BPC supports a meet and greet social on the first day of the annual meeting to allow travel award fellows to meet their cohorts, past cohorts, mentors and the BPC members. The BPC also supports a Diversity Social near the end of the annual meeting where travel award checks are distributed and where travel award fellows can meet and network with other SICB members, including past and current executive committee members, program directors from the National Science Foundation and other invited guests. These activities were initiated at the 2011 annual meeting and continued through the 2017 meeting, and were very successful.

### SICB resources

The SICB leadership is very committed to broadening participation in the society and has many resources already in place that enable BPC activities to be implemented effectively. The BPC has retained its annual budget of $15,000. The SICB webmaster, Mr Birenheide, designs and maintains the webpage and online resources for announcements and travel award applications. The SICB has a very effective administrative team (Burke Associates Inc.) that organizes the annual meetings. They are instrumental in allocating rooms for the workshops and socials (along with the Program Officer), organizes food orders for the socials, manage the budget and print award checks. The elevation of BPC to the SICB Executive Committee ensures that all of these activities will be retained in the future. These resources are at the disposal of the BPC who are a dedicated group of volunteer members that have a vision for broadening participation within the Society. The BPC recognizes the importance of creating and sustaining a diverse community of scientists, and is willing to commit time and energy to ensure that our goals are not only embraced but also achieved.

### SICB BPC objectives

**Fig. 1.**
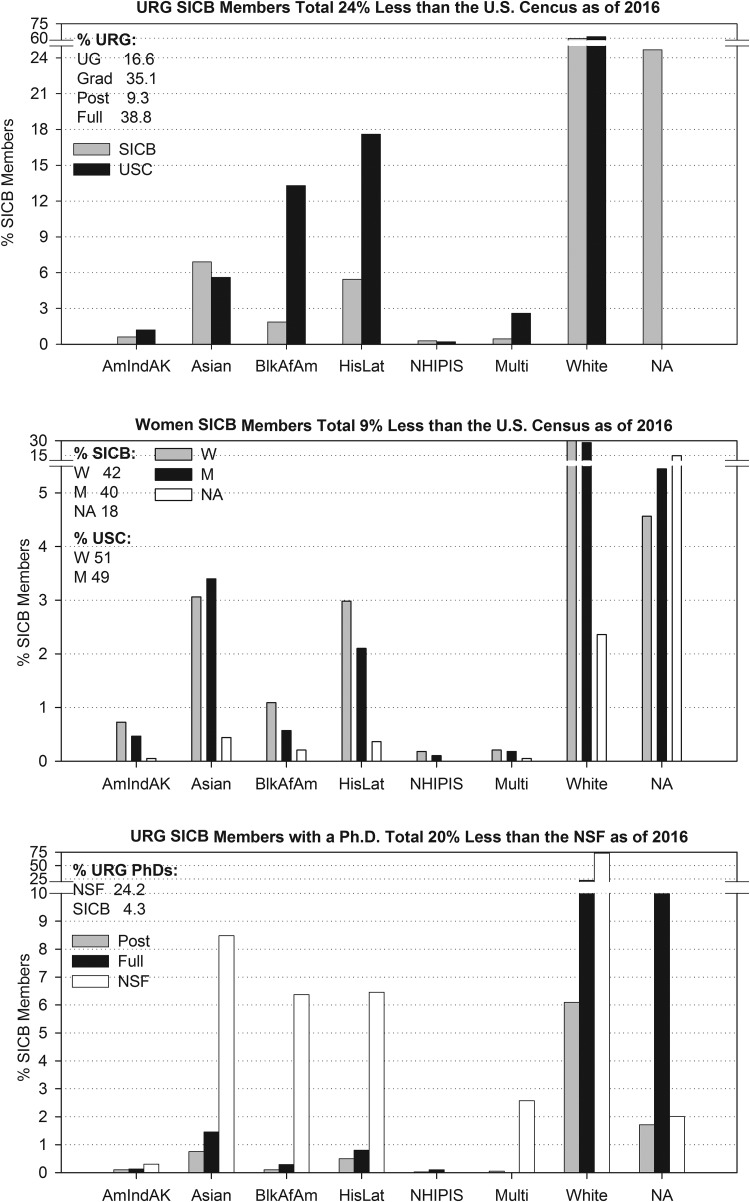
Disparity in SICB membership relative to the US Census and NSF PhD holders by ethnicity, gender and member level. SICB is comprised of 17% URG members, 4.3% of which hold Doctoral degrees, 42% women members and 8.2% are from URGs. Total 2016 membership is 3855. URG ethnicities: AmInAK, American Indian and Alaska Natives; BlkAfAm, Black and African Americans; HisLat, Hispanic and Latino; Multi, more than one ethnicity; NHIPI, Native Hawaiian and Pacific Islanders. Member level: Emer, emeritus; Full, faculty; Grad, graduate; Post, postdoctorate; UG, undergraduate; Gender: F, female; M, male. NA, not answered. Census: NSF, 2014 NSF census of PhD holders; USC, 2015 US population census.

The main goal of the BPC is to create a culture where members from URGs have access to a number of resources, such as mentors, workshops, funding for travel to annual meetings and a sense of community within the society. By building upon SICB’s base of URG members, immediate feedback can be obtained on the needs of those members who are currently at different stages of their careers (graduate students, postdoctoral fellows, junior faculty and senior faculty). The BPC can assess what resources fit the needs for each level of our URG member pool, and reinforce SICB’s ability to ensure their success. The BPC census of SICB composition has indicated that the URG numbers show a dramatic decline at the level of postdoctoral and assistant professors (Fig. 1); yet causes for this decline are not apparent from these data. Our objectives include tracking these data to attempt to identify and address as many of the causes as possible, and establish long-term solutions that will enable postdoctoral and assistant professor members to persist and grow within the SICB.

Eight BEST principles were identified as most successful in recruiting and retaining URG in other professional societies and in STEM fields (Wilson and Haynes 2002; Pandya et al. 2007; Payton et al. 2012). The goals of the BPC align closely with the BEST principles: 1) institutional leadership, 2) targeted recruitment, 3) engaged faculty, 4) personal attention, 5) peer support, 6) enriched research experience, 7) bridging to the next level and 8) continuous evaluation. Pandya et al. (2007) identified one pervasive need—financial support—that can easily be addressed by scientific societies. The BPC goals align well with these design principles and have implemented activities that address some of these, which include: 1) provide support for attending our annual meeting in the form of travel awards, which will promote and sustain a URG cohort that in turn will be our future leaders in the society and beyond; 2) increase awareness of broadening participation and building community by promoting events at the annual meeting (socials and workshops); 3) offer workshops that address issues specific for URG members (i.e., career development, leadership, teaching and outreach); 4) recruit new URG members and promote the society to other societies in which SICB members are involved and 5) have measurable outcomes that can be used for assessment of BPC goals. Positive outcomes will drive the direction and evolution of future CBP objectives (see Table 1 for current objectives). We will maintain or enhance those activities that we find are successful and modify those that are not working until the percentage of URG members mirrors that of the US population census.

**Table 1.** Objectives of the BPC

Objectives	Outcomes
1 + 2) Initiate a URG cohort and community that will be sustained throughout the career at all levels of membership. Addresses BEST #3–5.	Continue support for attending annual meetings, offer social events that bridge new and loyal URG members.
3) Provide leadership and professional training that sustains beyond their involvement with SICB. Addresses BEST #1, 6–7.	Offer workshops in best research practices such as grant writing, time management, leadership, funding opportunities.
4) Recruit members and promote the society to other organizations serving URG in the biological sciences relevant to the interests of the society. Addresses BEST #2–3.	Individual members who attend meetings such as AISES, SACNAS, MARC, AGEP and McNair can promote the benefits of being an active and diverse SICB member.
5) Determine whether the BPC initiatives are increasing diversity within the society and beyond. Addresses BEST #8.	Continually assess each aspect of the program, revise and redirect (if needed) the goals and objectives of the program.

*Notes*: AISES, American Indians in Science and Engineering; SACNAS, Society for the Advancement of Chicanos and Native Americans in Science; MARC, Maximizing Access to Research Careers; and AGEP, Alliances for Graduate Education and the Professoriate.

The current BPC goals align with prior key objectives that were identified as most successful in recruiting and retaining URG in other professional societies and in STEM fields (Wilson and Haynes 2002; Pandya et al. 2007; Payton et al. 2012).

## SICB BPC activities and assessment

### Demography of SICB

**Fig. 2.**
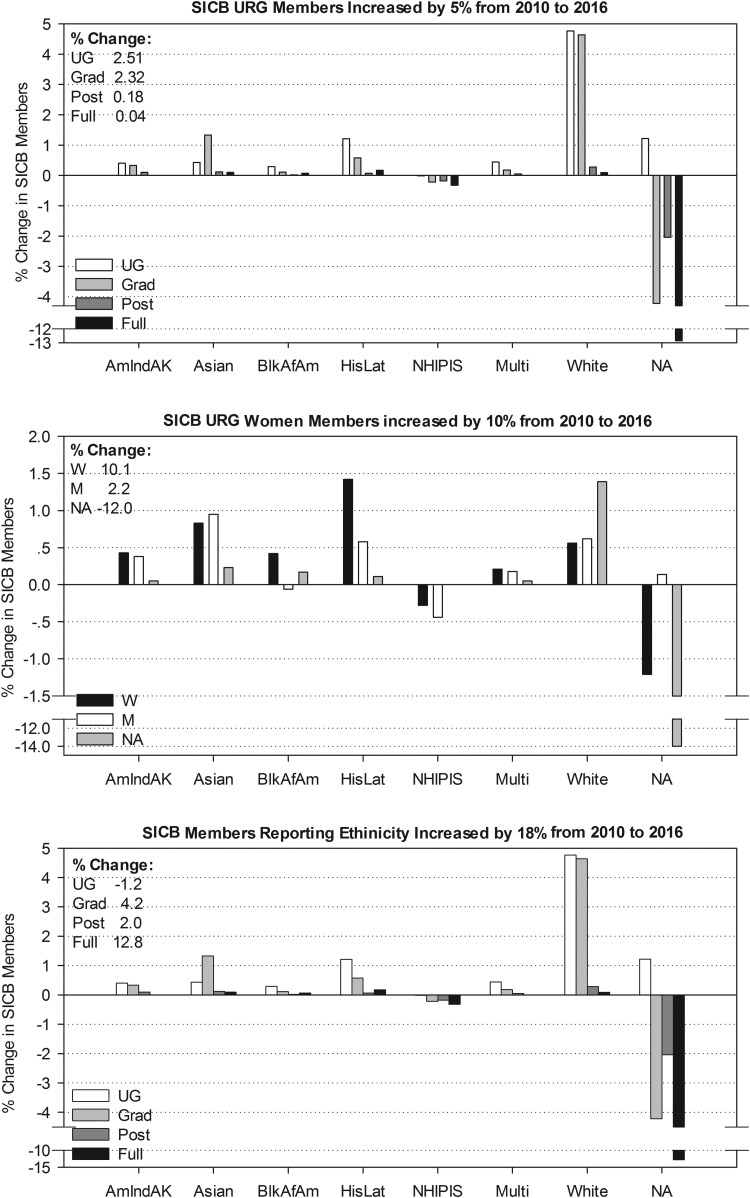
Effects of BPC activities on SICB membership from 2010 to 2016. URG members increased from 11% to 16%, women members increased from 32% to 42% and the member self-identification increased from 60% to 75%. Abbreviations as in Fig. 1.

SICB URG member gains was measured by implementing a mechanism to collect and assess self-identification of ethnicity, gender and disability (added in 2011) on annual membership (2010–2016) and meeting registration forms (2009–2016). This was a critical step in determining the ethnic, race, gender and disability makeup of our membership compared with 2010 US Census and NSF Doctoral Degree-Holder demographics (NSF 2009; US Census 2010). As of the 2010 annual conference, SICB had 2373 members, of whom 11.0% were from URGs, 49.3% were white (39.7% did not self-identify ethnicity), with 32% women members (30% left gender blank). As of the 2016 annual conference, SICB membership increased to 3855 members, an increase of 62% from 2010 (Fig. 1). URG members increased to 16.2%, with an increase in white members to 59.8% (Fig. 2). Women members increased to 42%, while men members increased by 2% (Fig. 2). Thus, there was a sharp decrease in the percentage of members that failed to self-identify ethnicity (down to 24.7%) and gender (down to 18.1%; Fig. 2). Associated with the 15.0% increase in self-identification is a 10.5% increase in white and a 5.2% increase in URG members (Fig. 2). Even with these remarkable gains, URG membership within the society is only 16.2%, essentially half the 37.9% reported in the 2015 US Census (US Census 2015). SICB members reporting a disability is 1.5% compared with 8.5% of Doctoral Scientists in biological, agricultural and environmental life sciences (ages 16–64 years) of the general population in the United States (NSF 2014; US Census 2015).

Comparing the current membership of SICB to that of the 2015 US Population Census indicates that SICB is doing well attracting some URG groups (Fig. 1). Asians and Native Hawaiian/Pacific Islanders are the only URGs currently in SICB where the percent of members exceeds that of the population census (by 23.2% and 42.7%, respectively) (US Census 2015). Hispanic/Latino and American Indian/Alaskan Natives members together comprise less than or half of the US population (31.0% and 51.9%, respectively, SICB/US%), with Black/African Americans members totaling only 14.0% of the US population (SICB/US%). In 2016, SICB added a new category called “multiple ethnicities” to which 0.44% of members selected in lieu of selecting an ethnicity. This made it easier for some members with multiple ethnicities to self-identify (more than one group can be selected); however, this decreases the precision of our assessment. The percentage of white SICB members is slightly less than the US Census (97%, SICB/US%). Women members number slightly more than men, 42.3% and 40.1%, respectively, which are proportionately similar to the US Census (50.8% women, 49.2% men). However, unless those 18.1% of members that leave gender blank self-identify, it will be unknown whether the gender ratio in SICB is truly equal to that of the US Census (US Census 2015).

SICB Membership consists of doctorate holding members that are Full (35% including 3.6% emeritus) and Postdoctoral (9.3%), with students comprising about half of the members: graduate (35%) and undergraduate (16.7%) (Fig. 1). Interestingly, most of the emeritus and nearly one-third of full members fail to self-identify ethnicity (79.3% and 28.4%, respectively) compared with postdocs (18.3%), graduate students (16.4%) and high school students (14.3%). On the other hand, undergraduate students (25.4%) fail to self-identify nearly as often as full members. SICB is doing well at attracting graduate students and full members, but is only retaining approximately 26.3% of the graduate student members as postdoctoral members. This may be due to the lack of support for postdoctoral members to attend meetings. Attracting more postdoctoral researchers, who can benefit from professional workshops and networking at the socials at the annual meetings, could lead to increased SICB members once they obtain faculty positions and bring their postdoctoral researchers and students. Sharing data about societal composition is a valuable resource for all professional societies to measure their efforts in the recruitment and retention of URG (Freehill and Ivie 2013). However, we were unable to find other scientific societies with similar membership data to compare our data too.

Relating the percentage of SICB members with Doctorates to the NSF Census of Doctoral Holders may be the more appropriate standard with which to compare the demography of a scientific society. Only 4.4% of URG SICB members hold a doctorate degree compared with 21.6% URG Doctoral Scientists reported in the 2014 Census of Doctoral Scientists in biological, agricultural and environmental life sciences in the United States (NSF 2014). SICB Full members are comprised largely of faculty in the academy, thus this may indicate a lack of successful recruitment of URG to the academy, or low URG recruitment to professional societies. URG SICB members with doctorates are at a lower level than NSF census Doctorate Holders in all ethnic categories: Hispanic/Latino with Doctorates (1.3%) in SICB compared with NSF Census Doctorate holders (6.5%); Asians with PhDs (2.2%) in SICB compared with NSF Census Doctorate holders (8.5%); Black/African Americans with PhDs (0.42%) in SICB compared with NSF Census Doctorate holders (6.4%) (NSF 2014). American Indian/Alaskan Native SICB members with Doctorates (9) are most similar to NSF census Doctoral Holders (0.23% versus 0.30%, respectively) because their populations are relatively low nationally. White SICB members with Doctorates comprise a third that of NSF Doctoral holders (29.3% versus 73.0%, respectively; NSF 2014).

With respect to leadership, SICB has had only nine women and no minority presidents since its inception in 1890, which means that 92% of past presidential terms have been white men (117 terms). In addition, few URG members are holding leadership or divisional positions within SICB. The reason for this lack of leadership may be partly due to the low number of URG SICB members with Doctorates, particularly at the full professor level. Having URG member representation on the executive committee would provide role models within the society, and present a collective societal voice representing diversity and inclusion for all scientists.

The Travel Award Program started with the 2011 annual meeting and has been successful in other scientific societies as well at SICB (Wilson and Haynes 2002). URG Members from all levels can apply for up to $500 to support travel to the annual meeting. As a mechanism to guide future efforts of the BPC to broaden participation within SICB, applicants are asked to state their career goals, describe two challenges to being a member from an URG in science and suggest workshop topics for the next annual meeting. The BPC was able to fund 86.6% of applicants in 2011, 53.6% of applicants in 2012, 91.6% of applicants in 2013, 92.5% of applicants in 2014, 58% of applicants in 2015 and 33% of applicants in 2016. Hispanic/Latino members received slightly more than half of the Travel Awards, followed by White Women and Black/African American members (Table 2). In 2011, more members were supported at a smaller amount than requested in order to fund most of the applicants. Many of the travel fellows verbally told BPC members that they would not have been able to attend without this funding. In 2012, most members requested the maximum amount stating that they would unlikely attend without full funding. Thus, fewer members were funded but with a higher level of support. In 2013, the deadline was moved earlier in the year to allow applicants to receive notification of the award before the registration deadline. Applicants requested the earlier deadline so they would know whether they had the funds to attend the meeting before registering for the meeting, however several members missed the earlier deadline. In 2014, BPC members Cheryl Wilga, Michele Nishiguchi and Brian Tsukimura were awarded an NSF Conference Grant for $25,000 to fund two SICB Broadening Participation workshops, URG workshop panelists and URG members at the annual meeting (IOS-1362663). As a result, 64 additional members were funded (92.5% of applicants). In 2014–2016, postdoc and junior faculty URG members were given priority to BPC travel funds in an attempt to increase the low member attendance at those levels. Overall Travel Awards were fairly equally spread out among undergraduate, doctoral and postdoctoral members, with a smaller percentage going to master student and assistant professor members. Two-thirds of the Travel Award recipients were women.

**Table 2.** Funded travel award demographics from 2011 to 2016 (176 in total)

Ethnicity	Percent	Level and gender	Percent
Hispanic/Latino	55.9	Asst. Professors	7.3
Asian	8.5	Postdoctorates	20.9
Black/African American	13.0	PhD Students	28.8
American/Alaskan Indian	4.0	MS Students	15.8
Native Hawaiian/Pacific Islanders	5.1	UG Students	26.0
White women	13.6	Females	71.8
		Males	32.8

The BPC offers two Professional Development Workshops at each annual meeting starting with the 2011 meeting. This is a main feature of broadening participation efforts in other scientific societies (Wilson and Haynes 2002). Workshops are chosen from the most requested topics suggested by the Travel Award Fellows. All SICB members are invited, and every workshop thus far has been successful with a mean of 74 members attending (range 30–100) (Table 3). One BPC workshop focuses on professional development for graduate students and the other focuses on faculty and postdocs. Of the 26 workshop hosts from 2011 to 2016: 16 were from URGs; 18 were women; 16 were Full Professors; 2 were Associate Professors; 6 were Assistant Professors; 2 were NSF Program Officers; 6 were current or past Chairs of the BPC; 9 were BPC members and 3 were SICB Executive Committee members (President and Program Officer) (Table 3).

**Table 3.** Professional development workshops from 2011 to 2014

Year	Name, number of attendees	Hosts
2011	Balancing Life and an Academic Career	Greg Florant,^a^^,^^b^ Nora Espinosa^a^^,^^b^
2011	Issues facing new faculty	Denise Dearing,^b^ Peggy Biga,^b^ Hannah Carey, Michele Nishiguchi,^a^^,^^b^ Scott McWilliams
2012	Science is a Two-way street: Mentorship and the Mentee	Michele Nishiguchi,^a^^,^^b^ Billie Swalla (President Elect), Cheryl Wilga^a^^,^^b^
2012	Demystifying the Grant Application Process	Cheryl Wilga,^a^,^b^ Michele Elekonich and Bill Zamer (NSF Program Directors)
2013	Effective presentations skills	Manny Azzizi,^a^ Patricia Hernandez,^a^^,^^b^ Andrew Clark^a^
2013	How to negotiate your first job	Gregory Florant,^a^^,^^b^ Billie Swalla (President)
2014	Recruitment strategies to obtain a diverse and thriving lab and department	Rebecca Calisi-Rodriguez,^a^ Michele Nishiguchi,^a^^,^^b^ Cheryl Wilga^a^^,^^b^
2014	Writing grants and manuscripts in a timely manner	Heather Bleakley, Brian Tsukimura^a^^,^^b^ (Past Program Officer), Michele Nishiguchi^1,2^
2015	The academic juggling trick: how to effectively manage your time during the professoriate	Michele Nishiguchi^a^,^b^
2015	Don’t be such a scientist, part II: How to give dynamic and informative presentations	Jake Socha (SICB Public Affairs Committee)
2016	Integrate diversity awareness into science institutions	Kendra Greenlee,^a^,^b^ Michele Nishiguchi^a^,^b^

a URG member.

b BPC member.

The pre-meeting “Meet and Greet” Social is hosted by the current Chair of the BPC on the first day before the plenary lecture and was initiated in 2012. This social brings together travel award fellows and members of the BPC for networking and to establish a cohort that will eventually build community within SICB and increase retention and form lasting post-meeting relationships. The pre-meeting strategy is to create a cohort of members at each meeting that can reconvene throughout the meeting to share thoughts and impressions about the meeting. Appetizers and soft drinks are provided by SICB funds in an informal setting with an attendance of approximately 30–50 members each year.

The Broadening Participation Diversity Social that started with the 2011 meeting has also triumphed in increasing awareness of the committee and its activities. Here the BPC provides a spread of appetizers, invites guest speakers (BPC members, NSF Program Officers, Past and Current SICB Presidents and SICB Executive Committee Members), recognizes the BPC Travel Fellows, presents the travel awards and provides a comfortable friendly atmosphere for all members to mingle and chat. Attendance started in 2011 at around 100 members, with at least 300 in 2013, and approximately 200 members in 2014–2016. Our hope is that members from URGs feel more comfortable interacting with current and past executive committee and BPC members, NSF program officers, and other members in a smaller, intimate atmosphere and therefore become more involved in the society. This also provides past and future cohorts with a venue for informal networking with leaders in their fields and within the Society.

## Impact of BPC activities

**Fig. 3.**
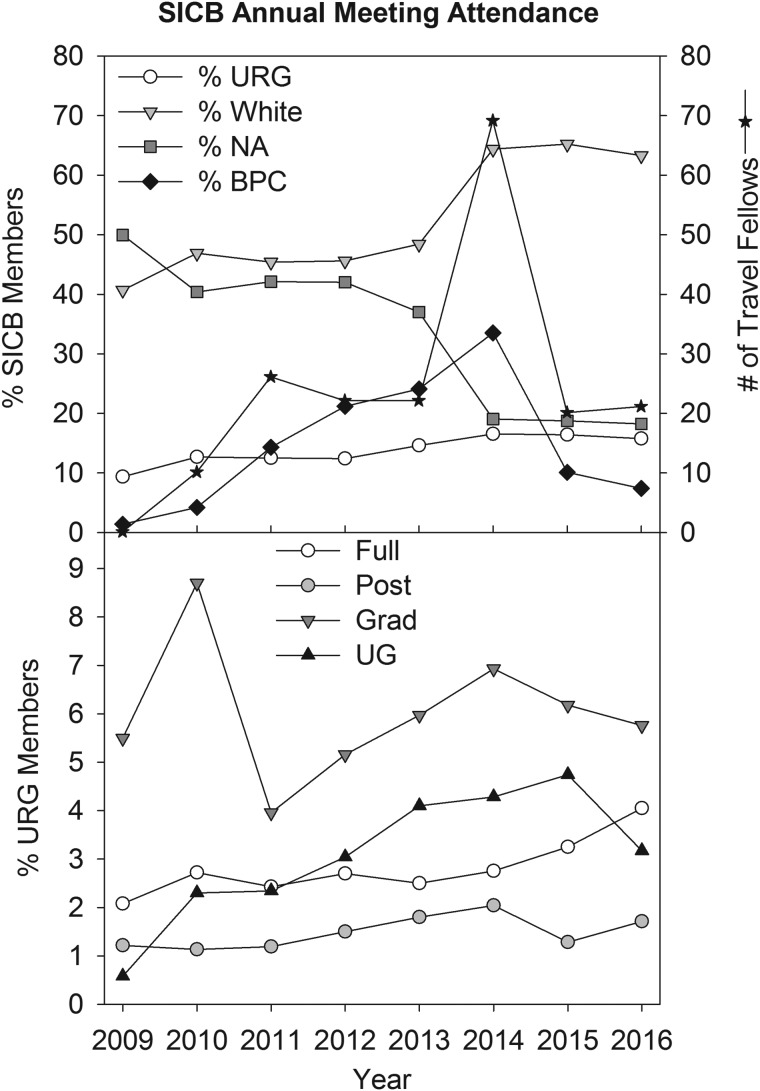
Effects of BPC activities on SICB member attendance at annual meetings. The top plot shows the percent of all members who attended the annual meeting by ethnicity, failed to self-identify and attended BPC activities. The number of URG members funded by travel awards is shown on the right axis (black asterisk). Note the sharp decline in the percent of members who fail to self-identify ethnicity (NA, gray boxes). Also note the steady increase in URG attendance with increased funding (open circles) and BPC professional development and social activities (black diamonds). The increase in 2014 attendance is due to the increase in BPC URG travel awards funded by a NSF Conference Grant. The bottom plot shows the demographics of URG members by level who attended the annual meeting. Note the steady increase in the percent of full and postdoctoral members (open and gray circles, respectively).

Over the past 5 years, the data show an increase in URG members (up by 5.2%), a decrease in members who do not report ethnicity and gender (down by 15% and 12%), and an increase in the number of members attending BPC activities (up to 33% by 2016) (Fig. 3). The striking decrease in the number of members who do not report ethnicity, especially over the last two years, suggests an increased awareness throughout SICB of the benefits of having a diverse membership. We are particularly pleased to see that postdoctoral member attendance is also steadily increasing. BPC efforts have succeeded in increasing diversity within SICB membership as well as those attending annual meetings as indicated by the steady increase in URG members and BPC activity attendance.

Travel awards have increased participation and enhanced attendance of URGs. Assessment of the BPC Travel Award program is very encouraging. Over the past 4 years, the BPC was able to fund 63.6% of the applicants, with 5% withdrawing their applications due to other funding being secured or inability to attend the meeting (Table 4). Funding for travel to annual SICB meetings is critical because 33% of the unfunded applicants did not attend the annual meeting (Table 4). Funding is also critical for continuing membership. Of those applicants that were funded (176 total), 38% remained in SICB as of 2016, with 41% leaving after 1 year of membership and only 14% leaving after 2 years of membership (Table 4). Nearly half of the awardees that leave SICB after at least 1 year are undergraduate students (44%), with graduate students the next largest group (33%). Thus, it appears that most Travel Award applicants decide after two meetings whether SICB fits their needs for a professional scientific society.

**Table 4.** Assessment of travel award applicants from 2010 to 2016

Travel award applicants = 176 total	%
Unfunded applicants of total	31.0
Unfunded applicants who also did not attend annual meeting	33.3
Withdrawn applicants of total	9.1
Funded applicants of total	63.6
Funded applicant status = 112 total	% funded
Remained SICB members as of 2016	37.9
Left SICB 1 year after funding	40.8
Left SICB 2 years after funding	14.4
Left SICB 3 years after funding	4.0
Left SICB 4 years after funding	2.9
Left SICB 5 years after funding	0.0
Received awards 2 years	10.5
Received awards 3 years	0.7
Received awards 4 years	0.9

To maintain or increase retention, it is imperative that a sense of community among the participants be developed (Hassoun and Bana 2001; Peckham et al. 2007; Jones et al. 2008; Koenig 2009; Xu and Martin 2011). Community can be developed through interactional engagement and social activities, such as pre-meeting and intra-meeting socials (Peckham et al. 2007; Jones et al. 2008; Xu and Martin 2011). URG members with increased interaction with executive committee and divisional leaders can develop a sense of community within SICB, especially if they feel that they are welcomed by the SICB’s membership (IPNs; Xu and Martin 2011). Mentoring, on informal individual and workshop scales, from peers and SICB leadership, can lower perceived barriers about engaging with senior members (Pandya et al. 2007; Koenig 2009; Tapia 2009; Payton et al. 2012; Wilson et al. 2012). In particular, mentoring early in one’s career is critical to enhancing professional success (Jones 2014).

Traditional presentations of the academy, and hence the professional society, are often culturally neutral ignoring participant perception or understanding of basic academy operations (Peckham et al. 2007; Jones et al. 2008). Shared experiences can often bring incongruent backgrounds together and initiate forming a sense of community. Our travel awardees form a natural cohort, which we bring together several times throughout the meeting at BPC socials and workshops so that they might find each other among the crowds, and to find how the meeting itself is a common experience.

We have increased retention through a supportive community formed through the BPC Travel Fellows, workshops and social programs. Continuing and bridging support to the next level of membership can be provided informally by SICB members, but also through BPC workshops. The Travel Fellows Program form a cohort facilitated by the socials and workshops that we hope will stimulate sustained interaction long after the annual meeting. The BPC Pre-meeting and Diversity Socials, open to the entire society, are informal and friendly places where these networks can easily be formed, increasing the potential for URG professional networking and mentoring (Xu and Martin 2011). The very successful and well attended BPC Diversity Social and workshops are already building community within the society and indicates buy in by member attendance.

SICB has already made a significant investment in broadening participation, not only financially, but also with a commitment by its members to increase URG diversity. Members are beginning to realize that increasing diversity will not only impact the Society’s ability to successfully facilitate different approaches to tackling problems within integrative biology, but will also help positively impact larger issues that develop throughout science and technology fields. A team composed of diverse members generates a greater breadth of solutions (i.e., “Grand Challenges in Organismal Biology—The need for synthesis” by Padilla et al. 2014). In order to facilitate and build a long-lasting community, SICB has provided the springboard in the BPC to initiate the welcoming of URGs into the society by striving for the goals stated in this paper. The SICB has also committed to a long-lasting and sustainable program that will recapitulate benefits from which all members of the society will gain, especially once SICB demographics match that of the US Census. SICB is a leader among long-lived scientific societies, and by succeeding in the increased awareness and participation of URGs, we will create a community of leaders who can bring their values, ideas and knowledge to improve broader scientific challenges that are of greater importance in today’s world.
